# U.S. hospital performance methodologies: a scoping review to identify opportunities for crossing the quality chasm

**DOI:** 10.1186/s12913-020-05503-z

**Published:** 2020-07-10

**Authors:** Kelly J. Thomas Craig, Mollie M. McKillop, Hu T. Huang, Judy George, Ekta S. Punwani, Kyu B. Rhee

**Affiliations:** IBM® Watson Health® Center for AI, Research, and Evaluation, 75 Binney Street, Cambridge, MA 02142 USA

**Keywords:** Hospital quality, Measures, Safety, Methods, Healthcare reporting, Ratings

## Abstract

**Background:**

Hospital performance quality assessments inform patients, providers, payers, and purchasers in making healthcare decisions. These assessments have been developed by government, private and non-profit organizations, and academic institutions. Given the number and variability in available assessments, a knowledge gap exists regarding what assessments are available and how each assessment measures quality to identify top performing hospitals. This study aims to: (a) comprehensively identify current hospital performance assessments, (b) compare quality measures from each methodology in the context of the Institute of Medicine’s (IOM) six domains of *STEEEP* (*safety*, *timeliness*, *effectiveness*, *efficiency*, *equitable*, and *patient-centeredness*), and (c) formulate policy recommendations that improve value-based, patient-centered care to address identified gaps.

**Methods:**

A scoping review was conducted using a systematic search of MEDLINE and the grey literature along with handsearching to identify studies that provide assessments of US-based hospital performance whereby the study cohort examined a minimum of 250 hospitals in the last two years (2017–2019).

**Results:**

From 3058 unique records screened, 19 hospital performance assessments met inclusion criteria. Methodologies were analyzed across each assessment and measures were mapped to *STEEEP*. While *safety* and *effectiveness* were commonly identified measures across assessments, *efficiency*, and *patient-centeredness* were less frequently represented. *Equity* measures were also limited to risk- and severity-adjustment methods to balance patient characteristics across populations, rather than stand-alone indicators to evaluate health disparities that may contribute to community-level inequities.

**Conclusions:**

To further improve health and healthcare value-based decision-making, there remains a need for methodological transparency across assessments and the standardization of consensus-based measures that reflect the IOM’s quality framework. Additionally, a large opportunity exists to improve the assessment of health equity in the communities that hospitals serve.

## Background

Today, hospital performance is increasingly important given growing demands to control healthcare costs [1, 2]. Hospitals are being reimbursed based on their ability to deliver high quality care and deliver value to patients [3], and patients are taking a more active role in their healthcare decisions [4]. Performance measurements are progressively being linked to reimbursement in pay-for-performance models [5]. Yet, quality metrics used in the measurement of value-based care may not optimally reflect the quality of care provided. Therefore, a need exists to balance quality initiatives with financial feasibility (i.e., value-based care).

Commonly used domains for understanding quality are the Institute of Medicine’s (IOM) framework (*safety*, *timeliness*, *effectiveness*, *efficiency*, *equitable*, and *patient-centeredness*; the acronym referred to as “*STEEEP*”). Using these domains may help balance quality with value for particular measures. Moreover, employing the domains of *STEEEP* may reduce variation in how care is delivered and practiced, revealing differences that exist across geographic, cost, and personal (e.g. racial) characteristics [6, 7].

IOM *STEEEP* proposes domains for quality care, but does not articulate specific quality indicators nor how to combine these measures to assess quality performance as a whole. Measures of performance are dependent upon the availability of data. Existing means of measuring hospital performance may include regulatory inspection or reporting, surveys, and statistical indicators which are often combined into composite scores. Although many measures exist, no clear consensus has been reached on which measures should be used for measuring hospital performance. For example, few common scores or standardized measures exist across the various national hospital ratings systems [8].

Yet, it is clear that better and worse methods of measuring hospital performance exist [9], such as consensus-driven and evidence-based indicators endorsed by the National Quality Forum (NQF) [10] and the Agency for Healthcare Research and Quality (AHRQ) [11]. Moreover the Donabedian framework can help guide how comprehensively quality is assessed across assessments using different performance measures. However, there are not clear guidelines for assessments to incorporate specific methodologies and appropriate measures to fit within the IOM’s *STEEEP* framework. Examining these aspects of existing hospital performance assessments are a first step toward developing more transparent and robust methods for determining how accurately and comprehensively hospitals provide quality care.

The purpose of the scoping review is to provide a comprehensive analysis of United States (US) methodologies used to assess hospital performance and their measures as they correspond to the IOM’s *STEEEP* quality framework. Using the *STEEEP* framework, quality domains and respective gaps were identified across currently available assessments using a systematic approach. Robustness (e.g. number of data sources and measures) and transparency of methodologies, to understand how measures were combined to assess hospitals, were evaluated. Additionally, in the context of informing policy to support value-based, patient-centered care, opportunities were identified for hospital assessments to “cross the quality chasm” [12].

## Methods

### Study design

A scoping review [13] was conducted in accordance with Preferred Reporting Items for Systematic Reviews and Meta-Analyses extension for Scoping Reviews (PRISMA-ScR) to identify studies that provide assessments of US-based hospital performance whereby the study cohort examined a minimum of 250 hospitals [14]. The review was designed to curate a comprehensive snapshot of recent and active methodologies regarding hospital performance in order to evaluate the current landscape. Therefore, inclusion criteria were limited to identifying published studies from 2017 to 2019 that included methodologies examining performance of 250 or more hospitals, which allowed for generalizable synthesis. Details of the methodology are provided in Additional File 1.

### Search strategy

A systematic search query of MEDLINE via PubMed and the grey literature was conducted to identify references published or available online between September 1, 2017 to September 1, 2019. This timeframe supports the identification of recently published hospital performance assessments.

### Screening process

Relevant references related to hospital performance assessment were screened and abstracted into standardized forms by independent dual review and conflict adjudication was provided by a third reviewer. Interrater reliability was determined by the kappa statistic [15].

### Data extraction

The following criteria were abstracted into standardized forms for synthesis and evaluation: *data source* including origin of data, data linkage, availability, type, sample size, and observation period; *cohort development* including inclusion/exclusion criteria and data pre-processing; *measure* (see below); and *score* including composite calculation.

### Measure characteristics

Beyond the data extracted from selected assessments as described above, specific measure or indicator characteristics were abstracted including name of measure, measure calculation, normalization, and explanation of why the measure was included, if any. Because measure characteristics were the focus, direct evaluation of the sensitivity of the measures was not conducted; however, data abstraction included how measures were chosen. Each measure was mapped, if possible, to categories within the Donabedian conceptual model of quality improvement which includes structural, outcome, and process categories, and the *STEEEP* framework for the domains of quality.

To determine if *STEEEP* mapped measures were supported by federal and non-profit organizations that lead consensus- and evidence-based measure reporting for healthcare quality, each measure was cross-referenced to AHRQ (prevention, inpatient, and patient safety quality categories) quality recommendations, and NQF endorsement.

## Results

### Summary of included assessments

**Fig. 1:**
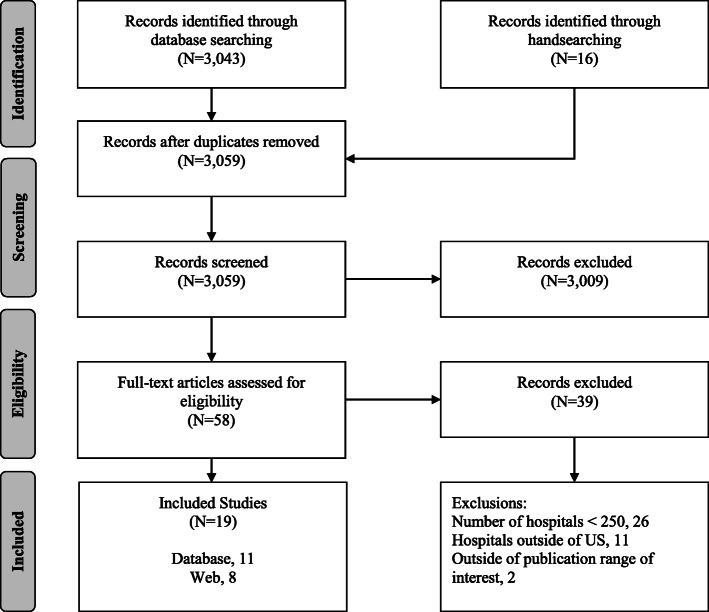
Results of the literature search, Preferred Reporting Items for Systematic Reviews and Meta-Analyses (PRISMA) flow diagram

From 3058 unique records screened, 19 hospital performance assessments described in the literature met inclusion criteria (Fig. 1). Of those studies, five de novo assessments [16–20], six evaluations of organizations’ ratings [21–25], and eight organizations providing assessments (with shorthand designation noted in brackets) were identified [26–33]: (1) Consumer Reports® Hospital Ratings [*Consumer Reports*], (2) Healthgrades™ America’s Best Hospitals [Healthgrades], (3) The Centers for Medicare & Medicaid Services (CMS) Hospital Compare [Hospital Compare], (4) IBM® Watson Health® 100 Top Hospitals® [IBM], (5) Island Peer Review Organization (IPRO), Why Not The Best? [IPRO], (6) The Joint Commission America’s Hospitals [Joint Commission], (7) Leapfrog Top Hospitals [Leapfrog], and (8) U.S. News and World Report Best Hospitals Procedures and Conditions [*US News*].

### Assessment methodologies overview

Four types of hospital assessments were identified: ranking, rating, listing, and evaluation-based studies. Ranking (IPRO, Hamadi et al. (2019) [16], Odisho et al. (2018) [19], Walkey et al. (2018) [34], Yokoe et al. (2019) [18]) assessments denoted a system by which all hospitals are arranged in order of ascending performance. Rating (*Consumer Reports*, Healthgrades, Leapfrog, *US News*; Al-Amin et al. (2018) [17]) assessments placed hospitals into relative quality groups. Listing assessments (Hospital Compare, IBM, Joint Commission) indicated hospital quality without comparison to other hospitals. Lastly, evaluation-based studies [20–25] provided critical examinations of hospital performance assessment methodologies from Hospital Compare [21, 23, 24, 35] and *US News* [21, 25].

Most of the assessments explained why specific measures were chosen for their particular methodology including the *Consumer Reports*, Hospital Compare, IBM, *US News*, and the de novo and evaluation-based studies. Reasons for including specific measures were wide-ranging, but centered on existing evidence that an indicator is associated with an endorsed quality outcome, such as mortality. Clear descriptions for why specific measures or indicators were chosen were not identified for Healthgrades, IPRO, Joint Commission, and Leapfrog.

Rather than addressing overall hospital performance, some studies assessed specific quality domains such as patient safety (e.g., surgical site infections [18], surgical procedures [20]), effectiveness (e.g., 30-day readmission [20]; 30-day mortality [34], and patient-centeredness (e.g., Hospital Consumer Assessment of Healthcare Providers and Systems (HCAHPS)) measures [17].

Summary information about the data sources, cohort development, scoring, and model performance across assessments can be found in Additional File 2.

### Performance measures

The kappa statistic for interrater reliability of data extraction was 0.69, including both Donabedian categorizations (e.g., structure, process, outcome) and *STEEEP* framework mapping. For simplicity of comparisons and to provide a subgroup analysis, this section will focus on the following eight organizations that provided overall hospital performance (i.e., reported and assessed information in more than one quality domain): *Consumer Reports*, Healthgrades, Hospital Compare, IBM, IPRO, Joint Commission, Leapfrog, and *US News*.

**Fig. 2:**
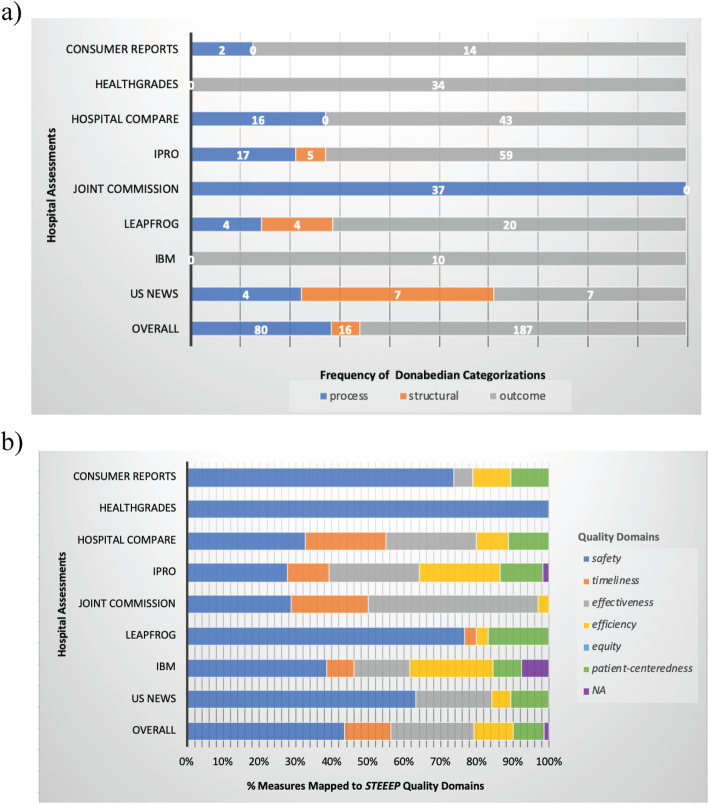
Frequency of (**a**) Donabedian categorizations and (**b**) percentage of STEEEP measures per assessment

Most performance assessments used primarily outcome (*n* = 187) and secondarily process-driven indicators (*n* = 80) while three (IPRO, Leapfrog, *US News*) also included structural-based measures (*n* = 16) to assess quality according to the Donabedian conceptualization (Fig. 2a). Three assessments did not use multiple concepts in their methodologies; Healthgrades and IBM exclusively reported outcome measures while the Joint commission methodology was limited to process measures.

Within the *STEEEP* quality framework, all assessments contained *safety*, five used *timeliness*, seven discussed *efficiency*, six used *effectiveness*, none explicitly reported *equity*, but five conducted risk- or disease severity-adjustments in models of other quality domains to address an equity-related issue (e.g., effectiveness and safety: race-adjusted mortality rate), and six included *patient-centeredness* indicators.

Across the assessments, measures were mapped (some to more than one domain); *safety* indicators (*n* = 168) were most commonly identified followed by *effectiveness* (*n* = 88), *timeliness* (*n* = 49), *efficiency* (*n* = 42), *patient-centeredness* (*n* = 33), and *equity* (*n* = 10) (using adjustments for equity-related variables) measures (Fig. 2b). Figure 2 summarizes the Donabedian conceptualization and *STEEEP* framework mapping of identified quality measures across assessments. Notably, some structural measures were unable to be mapped to *STEEEP* (e.g., adjusted operating profit margin, hospital-specific designations, percent of Medicare beneficiaries of all ages with diabetes or heart disease, and programs data).

**Fig. 3:**
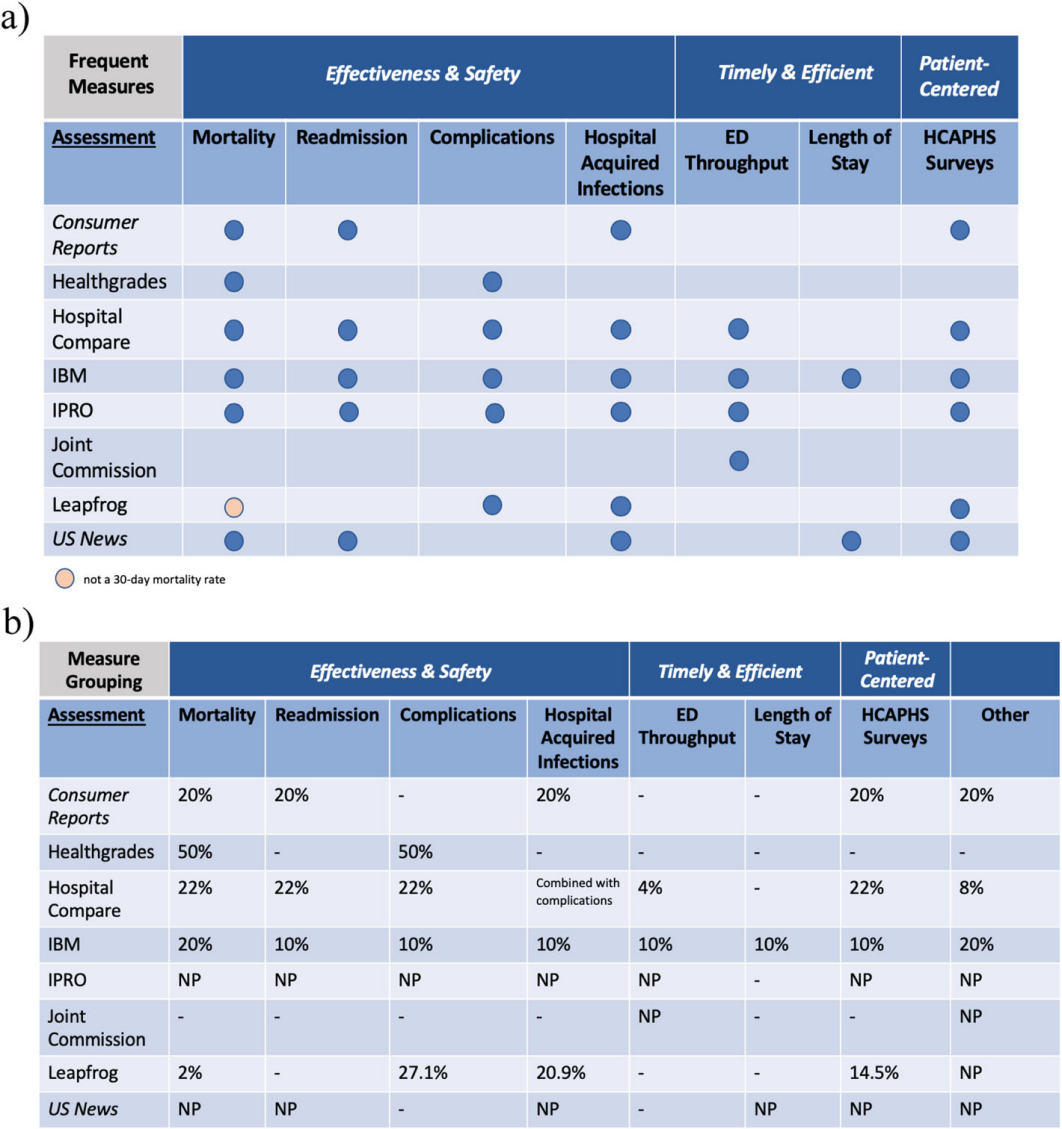
Frequent quality domain (**a**) measure overlaps and (**b**) comparison of their weights among assessments **a**) Abbreviations: ED, emergency department; HCAHPS, Hospital Consumer Assessment of Healthcare Providers and Systems. **b**) Descriptions of “Other” across assessments. *Consumer Reports*: Other, efficient use of imaging process measures; Hospital Compare: Other, 4% efficient use of imaging and 4% effectiveness of care process measures (e.g., patients assessed and given influenza vaccination; percentage of patients who left the ED before being seen; percentage of patients who came to the ED with stroke symptoms who received brain scan results within 45 minutes of arrival; percentage of patients receiving appropriate recommendation for follow-up screening colonoscopy; percentage of patients with history of polyps receiving follow-up colonoscopy in the appropriate timeframe; percent of mothers whose deliveries were scheduled too early (1-2 weeks early), when a scheduled delivery was not medically necessary; percentage of patients who received appropriate care for severe sepsis and septic shock; patients who developed a blood clot while in the hospital who did not get treatment that could have prevented it; percentage of patients receiving appropriate radiation therapy for cancer that has spread to the bone). IBM: Other, 10% operating profit margin (no mapping) and 10% adjusted inpatient expense per discharge for efficiency. IPRO: Other, weight not provided for timely and effective 1) stroke care (thrombolytic therapy, antithrombolytic therapy by end of hospital day 2, VTE prophylaxis, discharged on antithrombolytic therapy, anticoagulation therapy for atrial fibrillation/flutter, discharged on statin medication, stroke education), and 2) blood clot prevention and treatment (VTE prophylaxis, intensive care unit VTE prophylaxis, incidence of potentially preventable VTE, anticoagulation overlap therapy, unfractionated heparin with dosages/platelet count monitoring, warfarin therapy discharge instructions; safety, early elective delivery rates; efficiency, spending per Medicare beneficiary and health care costs; structural HIT measures and imaging for efficiency and safety; efficiency, population health and utilization costs; structural measures from county health rankings data on health factors and health outcomes related to preventive care for safety. Joint Commission: Other, weight NP for process measures. Timely and effective 1) stroke care (thrombolytic therapy, antithrombolytic therapy by end of hospital day 2, VTE prophylaxis, discharged on antithrombolytic therapy, anticoagulation therapy for atrial fibrillation/flutter, discharged on statin medication, stroke education; assessed for rehabilitation, VTE discharge instructions, and 2) blood clot prevention and treatment (VTE prophylaxis, intensive care unit VTE prophylaxis, incidence of potentially preventable VTE, anticoagulation overlap therapy, unfractionated heparin with dosages/platelet count monitoring, warfarin therapy discharge instructions; safety, early elective delivery rates; safety and effectiveness of antenatal steroids; safety and effectiveness for inpatient psychiatric services (admission screening, physical restraint, seclusion, and justification for multiple antipsychotic medications); safety and effectiveness of preventive care for influenza immunization, tobacco use (screening, treatment provided or offered, treatment provided or offered at discharge), hearing screening, alcohol use (screening, brief intervention provided or offered, or other drug use treatment provide or offered at discharge); effectiveness of exclusive breast milk feeding; surgical care effectiveness and safety of urinary catheter removal and antibiotics within one-hour before first surgical cut; safety and effectiveness, children’s asthma care, home management plan of care; and timely acute myocardial infarction measures (fibrinolytic therapy within 30 minutes and primary percutaneous coronary intervention received within 90 minutes). Leapfrog: Other, 23.1% safety practice process measures (leadership structures and systems; culture measurement, feedback, and intervention; identification and mitigation of risks and hazards; nursing workforce; hand hygiene) and 11.5% HIT (computerized physician order entry and bar code administration) safety, timeliness, and efficiency. Notably, the weights provided by Leapfrog only sum to 97.3% rather than 100%. *US News*: Other, weight NP for process measures on effectiveness (patient flu immunization and worker flu immunization) and safety (noninvasive ventilation and transfusion); outcome measures on patient-centeredness and safety (discharge to location other than patient’s home); structural safety measures related to information on board certifications and specialties, number of patients (volume), nurse staffing, number of intensivists, and transparency (reporting of performance). Abbreviations: ED, emergency department; HCAHPS, Hosptial Consumer Assessment of Healthcare Providers and Systems; HIT, health information technology; NP, not provided; VTE, venous thromboembolism; -, not an included measure

**Fig. 4:**
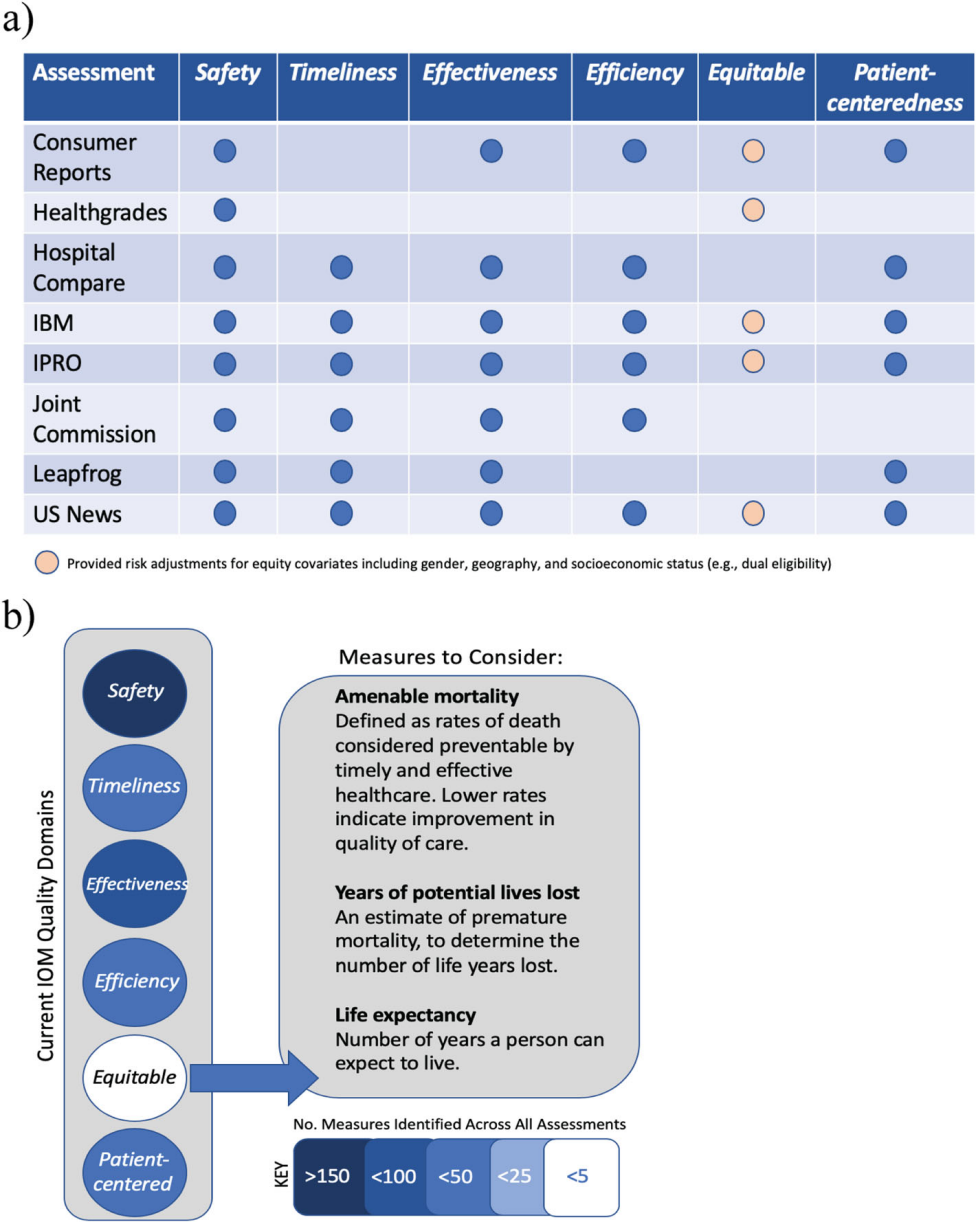
Identifying hospital performance gaps in *STEEEP* across assessments (**a**) Summary of quality measures mapped to *STEEEP* across assessments. No specific measures for equity were identified, but risk adjustment may have been done using equity-based variables; (**b**) *Equity* measures represent an opportunity for improvement; additional considerations for new measures in quality are suggested. The color range key represents the number of measures identified across all assessments where the darker color of blue indicates a higher frequency. While these measures for equity consideration could also be considered measures of effectiveness, we suggest that the influence of variables such as race, ethnicity, gender, socioeconomic status, and geography (at a minimum) on health and healthcare delivery outcomes could be further examined here. This is an opportunity for stakeholders to evaluate relationships among various types of inequality with the hopes to identify mechanisms and possible interventions to promote health equity in their communities

Common themes among process and outcome measures mapped to the *STEEEP* framework were identified along with their respective weights to determine hospital scoring (Figs. 3 and 4). Large overlap or similarity of identified measures occurred in the following themes: *safety* and *effectiveness* domains included mortality, readmission, complications, and hospital acquired infections (HAIs); *timely* and *efficient* care regarded emergency department (ED) throughput and length of stay (LOS); and lastly, *patient-centeredness* was limited to patient experiences summarized by HCAPHS survey data (Fig. 3a). The weighting of these frequent *STEEEP* quality indicators varied widely across assessments or was not provided (Fig. 3b). Mortality weighting ranged from 2 to 50%; readmissions indicators contributed to roughly 20% of the score when weighted; complications weighting ranged from 10 to 50%; ED throughput weighting range was lowest with 4–10%; LOS was only weighted at 10% by one assessment; and HCAPHS survey data contributed 10–22% of the scoring. Figure 3b details the weights provided for other measures that were not commonly identified across assessments to demonstrate transparency of scoring, where possible.

With *safety* and *effectiveness* as overt priorities in hospital performance outcomes, 30-day mortality and 30-day readmission rates were commonly identified with the exception of Joint Commission and Leapfrog assessments; notably, Leapfrog used death rate of surgical inpatients with serious treatable conditions as a measure of mortality. These 30-day effectiveness of care measures identified varied in their risk- and severity- adjustments, as did patient conditions (acute myocardial infarction, chronic obstructive pulmonary disease, heart failure, pneumonia, and/or stroke) as components of these composite outcomes. Harm outcomes were also frequently represented across assessments (except Joint Commission) including medical and surgical complications and HAIs. Medical complications were occasionally grouped with HAIs when the AHRQ patient safety indicator (PSI) 90 was used; other medical complication measures examined pressure ulcer rates, iatrogenic pneumothorax rates, in-hospital falls and trauma, and venous thromboembolism (VTE) incidence. Surgical complications varied greatly, but the most frequently identified measures related to hip fracture treatment, hip and knee replacements, and postoperative respiratory failure and wound dehiscence rates. HAIs measures commonly included catheter-associated urinary tract infections (CAUTIs), *Clostridium difficile* (C. diff) infections, central-line associated bloodstream infections (CLABSIs), methicillin-resistant *Staphylococcus aureus* (MRSA) infections, severe sepsis and shock, and surgical site infections (SSIs).

*Timely* care outcomes that reduce wait times or harmful delays and *efficient* care outcomes that reduce cost and unnecessary resource utilization, as adapted from AHRQ and CMS definitions, were identified as common *STEEEP* domains. Four (Hospital Compare, IBM, IPRO, Joint Commission) assessments focused primarily on ED throughput measures, and LOS was examined by two assessments (IBM provided severity-adjusted LOS when compared to unadjusted LOS by *US News*). ED throughput measures considered median times from ED arrival to ED departure for both admitted and discharged ED patients as well as admit decision time, time to pain management, time to fibrinolytic therapy, and patients left without being seen.

Patient experience (*patient-centeredness*) outcomes were identified in most assessments except Healthgrades and Joint Commission. The results were derived from survey questions using HCAPHS data; most were a composite of multiple categories related to communication from provider, patient-provider relationships, receiving help when needed, controlling pain, cleanliness of room, quietness of room, likelihood to recommend hospital, and overall patient experience.

*Equity*-based measures were not stand-alone metrics to demonstrate the remediation of differences in the quality of health and healthcare across different populations in the communities that hospitals serve. Identified *equity* measures included risk- and disease severity-adjustments for covariates such as gender, geography, and socioeconomic status (e.g., Medicare/Medicaid dual eligibility as a proxy) and were used by five assessments (*Consumer Reports*, Healthgrades, IBM, IPRO, *US News*) in LOS, mortality, complications, and/or post-surgical infection measures (Fig. 4a).

Measure developers, both government and non-profit, provide endorsements using consensus and evidence-based review. These recommended measures allow comparisons of performance to recognized standards for the improvement of care and outcomes. Identified measures were mapped to AHRQ and NQF endorsements (Table 1). Using standardized quality indicators from the AHRQ as benchmarks, patient safety indicators were included by all assessments except the Joint Commission. AHRQ inpatient indicators were used by all assessments except IPRO, Joint Commission, and Leapfrog. AHRQ prevention measures were only used by IPRO. Upon examining NQF endorsements of AHRQ measures, all assessments used at least one measure endorsed by NQF in each AHRQ category.

**Table 1:** *STEEEP* quality domains according to the Donabedian framework with national endorsement mapping

Assessment	Donabedian Category (measure count)	*STEEEP* Quality Measures	AHRQ Indicator(s)^b^
*Consumer Reports*	Outcome (14)	*Effectiveness, Equitable*^*a*^*, Patient-centeredness, Safety*	Inpatient, Patient Safety
	Process (2)	*Efficiency, Safety*	None
Healthgrades	Outcome (34)	*Equitable*^*a*^*, Safety*	Inpatient, Patient Safety
Hospital Compare	Outcome (43)	*Effectiveness, Efficiency, Patient-centeredness, Safety, Timeliness*	Inpatient, Patient Safety
	Process (16)	*Effectiveness, Efficiency, Safety, Timeliness*	None
IBM	Outcome (10)	*Effectiveness, Efficiency, Equitable*^*a*^*, Patient-centeredness, Safety, Timeliness*	Inpatient, Patient Safety
IPRO	Outcome (59)	*Effectiveness, Efficiency, Equitable*^*a*^*, Patient-centeredness, Safety, Timeliness*	Patient Safety, Prevention
	Process (17)	*Effectiveness, Efficiency, Timeliness*	None
	Structural (5)	*Efficiency, Safety*	None
Joint Commission	Process (37)	*Effectiveness, Efficiency, Safety, Timeliness*	None
Leapfrog	Outcome (20)	*Patient-centeredness, Safety*	Patient Safety
	Process (4)	*Safety*	None
	Structural (4)	*Efficiency, Safety, Timeliness*	None
*US News*	Outcome (7)	*Effectiveness, Efficiency, Equitable*^*a*^*, Patient-centeredness, Safety*	Inpatient, Patient Safety
	Process (7)	*Effectiveness, Patient-centeredness, Safety*	None
	Structural (4)	*NA, Safety*	None

^a^, equity-based measures used risk- or severity-adjustments in other *STEEEP* measures; ^b^, at least one measure in AHRQ category was NQF endorsed. *Abbreviations*: *NA* not applicable, *NQF* National Quality Forum

## Discussion

Hospital performance is often assessed beyond the examination of quality measures, including financial health and employee health of the organizations being reviewed. This study intended to examine quality domains (i.e., *STEEEP*) and their use as part of hospital performance assessment, and identify relationships, if any, between the two. Coverage and weighting of measures mapped to the *STEEEP* framework varied across assessments, which indicates that there is limited consensus on how to best measure hospital quality. Moreover, disparate measures and methodological disagreement may foster cynicism and confusion [9] among stakeholders that include patients, providers, payers, purchasers, and policy makers. This does not mean quality assessments should be disregarded, but that they should be considered in the larger context of hospital performance.

Our identification of evaluation-based studies that critically examined assessment methodologies determined that ranked or rated hospitals do not necessarily associate with quality [21, 25]; high performing hospitals did not have better CMS-based outcomes compared to other low performers. A suggested reason for this difference is that performance may be skewed by factors not directly related to quality such as patient volume, where high-volume facilities had better ratings [23]. Moreover, a number of other hospital-level characteristics, such as academic tertiary care center status [35], have been associated with poor performance on CMS-calculated metrics [17, 35]. These examples demonstrate that no assessment methodology is perfect, but each has its own set of strengths to inform their intended audience for the improvement of care and clinical outcomes.

Process measures should reflect evidence-based practices that systematically improve care and prevent negative outcomes. Frequently identified process measures were primarily centered on the *effectiveness* of care; these protocols to reduce the variation in care and improve the safety and efficiency of healthcare delivery were focused on VTE prevention and treatment, communication practices related to education and discharge information for continued therapeutics, and preventive interventions including immunizations, screenings, and management of care. The majority of assessments included process measures in their methodology as they are tied to reimbursement, accreditation requirements, and/or state and federal mandates. However, providers are pushing for more outcome metrics specific to the patients they serve in addition to process metrics [36].

### Develop patient-centered outcome measures along the continuum of care

Understanding patient outcomes is pivotal to provide value-based care and allows the opportunity to refine and improve care [37]. The majority of identified measures focused on outcomes across *safety and effectiveness*; currently, less consideration is provided to improve *patient-centeredness*, which should be equivalently emphasized in hospital assessment. Patient experience outcome measures were self-reported using HCAPHS surveys. These data cover a range of interactions that patients have with the healthcare system including care received from their doctors, nurses, and staff. These are particularly important measures as positive patient experiences are related to better health outcomes including lower readmission rates [38]; moreover, HCAPHS scores are commonly tied to value-based reimbursement. However, measurable value is generated by improving patient outcomes with particular conditions across the comprehensive continuum of care, and may involve multiple specialties at numerous care sites rather than on individual patient encounters.

Hospitals’ value-based performance depends upon health and healthcare received by its patient population prior to and following the care delivered within hospital walls. Measure developers should aim to incorporate outcome-oriented and patient-centered viewpoints, using a combination of clinical, claims, and patient-reported longitudinal data rather than using one dataset from a single site at one snapshot of time in the patient’s receipt of care. A significant roadblock to implementing these types of measures is the inability to easily exchange healthcare data, a lack of interoperability. Notably, the U.S. Department of Health and Human Services have recently implemented two rules (from the Cures Act and MyHealthEData initiative) (Available from: https://www.hhs.gov/about/news/2020/03/09/hhs-finalizes-historic-rules-to-provide-patients-more-control-of-their-health-data.html) requiring both private and public entities to share information between patients and other parties in a private and secure manner. This access to health information will help resolve interoperability barriers when obtaining data required to support innovative patient-centered outcome measures.

### Gaps in measuring hospital quality: equity and efficiency

As patient-centered models and value-based payments systems gain support, hospitals will need to monitor and evaluate services received outside their walls for reporting purposes and effective care management from the patient perspective. As such, hospitals will (and should) be held accountable for health outcomes outside of their facilities in the communities they serve. Although addressing health equity is difficult and will not be solved by hospitals alone, hospitals play an important role in community health and should attempt to address population health concerns in their area; moreover, this will require the reconsideration of how hospitals are incentivized to provide care.

Monitoring of health inequities, observing differences in health between subgroups within their community, is essential to achieve health equity. Recently Hamadi et al. (2019) demonstrated that healthcare quality measurement does not adequately adjust for the differences in serving these communities including access and affordability, prevention and treatment, and avoidable hospital use and cost; the level of minority presence and hospital reimbursement policies influence referral region health rankings [16]. Unfortunately there is limited consensus on direct measures of health equity, but opportunities exist to examine the relationship between population groups that exhibit disparities in health and healthcare delivery outcomes. Ideally, equity-related considerations should be a part of every quality domain (i.e., the remaining *STEEP domains*). Focusing on recommended equity measures, or examining other equity-related measures, such as hospital workforce diversity, are places to begin to address health equity. Healthcare is built on a foundation of rapport and trust, and both are garnered in part when providers emphasize cultural and linguistic competency in health and healthcare decision-making for their patients. Quality of care for a community can be improved by building a diverse workforce that represents the community.

Increasingly, social determinants of health (SDoH) are being recognized as important proxy measures of health equity as well as supportive of value-based care. Yet across assessments we find only one (IPRO) broached health equity issues related to SDoH using access to care data and percent inadequate social support. Preliminary return-on-investment analyses [39–41] and policy recommendations [42, 43] prioritize collection of actionable SDoH factors such as education, food, and housing to reduce costs using targeted community-based interventions. There are several US datasets, mostly public survey-based surveillance data, that can be used to extract insights on diverse populations (e.g., racial, ethnic, sexual/gender minority groups) and to assess health equity and/or social determinants [44–47]. However in order to develop richer and more diverse SDoH datasets, incentives to track and share SDoH data are needed in order to better achieve health equity in the context of value-based care.

This viewpoint, that hospitals should use SDoH data for the purposes of quality assessment of health equity, re-orients the traditional role of the hospital from an institution designed to cure, to a health system supporting population health. Arguably, this is not a simple task given the highly fragmented nature of healthcare today and payment incentives. However, there are some steps hospitals can take. For example, most hospitals complete a community needs assessment every three years to help identify resource needs of their community and develop programming, of which many relate to SDoH variables such as education, food, and housing; these gaps in care are addressed directly with local stakeholders to improve healthcare. By addressing the needs of the community, hospitals are taking a primary role to improve the outcomes of the communities that they serve. In doing so, there is greater value by addressing SDoH, which are associated with substantial costs to the healthcare system.

The transition towards measuring patient-centered outcomes will also depend on how well healthcare and social care can address the needs of those most at risk for poor outcomes. Expanded consideration of equitable care should influence the design of future quality measures, which will require increased development and testing of prevention and equity indicators. Potential publicly available equity measures (e.g., amenable mortality, life expectancy, or potential lives lost) are discussed in Fig. 4b. However, community surveillance-based measures will require standardized, accurate, and secure data platforms to access a comprehensive view into the health of individual patients. Additionally, the use of disaggregated datasets, from multiple sources, to represent inequity dimensions (e.g., socioeconomic, demographic, or geographic factors) can facilitate data-driven policies, programs, or practices to advance equity [48–50].

The ability of hospitals to support their communities through wellness initiatives is dependent upon fiscal solvency. Community benefit planning, including expansion of health equity programs, requires investment strategies dependent upon operating margins, which are linked to performance-based incentives. Only one assessment (IBM) provided insight into the financial health of hospitals using an outcome measure of operating profit margins, which was not mapped to *STEEEP*. Until policy incentives are shifted, equity assessments will likely be de-prioritized as hospitals follow suit with CMS and other guidelines for reimbursement.

An additional gap identified was fiscal insight into operating efficiency, how hospitals balance the need for quality with cost. These types of measures are increasingly important with the trend toward value-based care. This need is reinforced by the fact that 8% of US hospitals are at risk of closing and 10% are considered fiscally weak [51]. Only two assessments examined outcome measures related to cost of care (case-mix and wage-adjusted expense per discharge, IBM; and several cost reports related to Medicare reimbursement among several diagnosis related groups, IPRO).

### Strategies to improve hospital performance assessment

Methodologies examining hospital performance were identified as limitations to many assessments in this review; the transparency and rigor of methodologies were frequently noted as low. Evaluation-based research of methodologies by peer-review to improve measure testing, model improvements, and scoring would be valuable for all assessments. Further, given the large quantity of data required to derive assessments; the use of data-driven analytics would increase study rigor. For example, a semi-supervised machine-learning algorithm applied to publicly-available quality measures for US hospitals provided a novel clustering and visualization method to identify differences in performance that are typically obscured by existing hospital rating systems [52].

Given the variety in types of hospitals examined, it is challenging to provide an apples-to-apples comparison for short-term acute care settings, so classification models to create more homogeneous hospital groupings would be advantageous. Additionally, artificial intelligence methods such as machine learning algorithms could be used to improve model performance, evaluate variables that are used to create hospital grouping classifications, or identify factors that are associated with high-performance.

### Strengths and limitations

This review has several strengths. First, this is a novel review that examines assessments targeting a wide range of audiences; while other articles have compared hospital ratings [8, 9, 53, 54], none have objectively evaluated methodologies related to the IOM quality framework. Secondly, a rigorous, scoping methodology was applied in our approach. An exhaustive literature search was conducted for the time limit, including an evaluation of grey literature and web-based content that prioritized sensitivity over specificity, revealing a lack of peer-review for all assessments; however, some assessments were more transparent in their methodology than others. An opportunity exists for critical evaluation, including assessment of risk model performance and information on measure reliability and validity, by external reviewers to support credibility and trustworthiness of hospital performance measures.

Our results should be interpreted in the context of a few limitations. This scoping review search was limited in scope and comprehensiveness because only two years of articles were reviewed; however, we felt this search limit (2017–2019) was necessary in order to assess the current landscape of hospital performance methodologies. Additionally, performance methodologies that examined less than 250 hospitals were excluded to provide more generalizable results and as such, some relevant studies may have been missed. Measure mapping to Donabedian categories and the *STEEEP* framework had moderate interrater reliability (0.69), but the research team interpreted the measures using IOM definitions. As such, there were instances of disagreement between Donabedian categorizations made by the assessments and our review as well as mapping to *STEEEP*. Similarly, it was challenging to map measures exactly to AHRQ indicators. Reviewers used a standardized approach for labeling, to be consistent when measure definitions were not exact fits, but the domain and intent of the measure was the same. An additional limitation was the dependence on publicly available information during the chosen time frame; some assessments had removed web content, did not have timely web content updates, or lacked transparency to identify abstracted information and gaps in data. Notably, our *US News* evaluation was limited to the Procedures and Conditions rating report, but their Best Hospitals Honor Roll includes the Specialty ranking methodology; this approach allowed a more equivalent comparison to the acute care hospitals being examined, as no other assessments evaluated specialty hospitals.

## Conclusions

There is a need for the standardization of consensus-derived quality measures that reflect the changing landscape of value-based care and patient-centered healthcare models. While *safety* and *effectiveness* were commonly measured quality indicators, there were less frequent uses of *efficiency* and *timeliness*, and no direct measures of *equity* were identified, only adjustments for covariates. Quality measure developers should consider *patient-centered* outcomes and include *efficiency* measures to assess cost and operating margins. Their impact on a hospital’s ability to support the expansion of equity-based programs, community-linked initiatives to address SDoH, and health disparities issues that greatly impact health and healthcare should be assessed.

## Supplementary information

**Additional file 1.**

**Additional file 2.**

**Additional file 3.**

**Additional file 4.**

**Additional file 5.**

**Additional file 6.**

**Additional file 7.**

**Additional file 8.**

**Additional file 9.**

## Data Availability

All supporting data has been provided in additional files.

## Abbreviations

AHRQ: Agency for Healthcare Research and Quality
CMS: The Centers for Medicare & Medicaid Services
ED: emergency department
HAIs: hospital acquired infections
HCAHPS: Hospital Consumer Assessment of Healthcare Providers and Systems
IBM: International Business Machines
IOM: Institute of Medicine
IPRO: Island Peer Review Organization
LOS: length of stay
NQF: National Quality Forum
PRISMA-ScR: Preferred Reporting Items for Systematic Reviews and Meta-Analyses extension for Scoping Reviews
*STEEEP*: *Safety, Effectiveness, Efficiency, Equitable, and Patient-centered*
VTE: venous thromboembolism
US: United States

## References

[1] Hilsenrath P, Eakin C, Fischer K. Price-transparency and cost accounting: challenges for health care organizations in the consumer-driven era. Inquiry. 2015;52:0046958015574981. 10.1177/0046958015574981. 10.1177/0046958015574981 PMC5813634 25862425

[2] Martin AB, Hartman M, Washington B, Catlin A (2019). National Health Care Spending In 2017: Growth Slows To Post-Great Recession Rates; Share Of GDP Stabilizes. Health Aff (Millwood).

[3] Elliott MN, Beckett MK, Lehrman WG, Cleary P, Cohea CW, Giordano LA (2016). Understanding the role played by Medicare's patient experience points system in hospital reimbursement. Health Aff (Millwood).

[4] Pomey MP, Ghadiri DP, Karazivan P, Fernandez N, Clavel N (2015). Patients as partners: a qualitative study of patients' engagement in their health care. PLoS One.

[5] Kahn CN, Ault T, Potetz L, Walke T, Chambers JH, Burch S (2015). Assessing Medicare's hospital pay-for-performance programs and whether they are achieving their goals. Health Aff (Millwood).

[6] Baicker K, Chandra A, Skinner JS (2005). Geographic variation in health care and the problem of measuring racial disparities. Perspect Biol Med.

[7] Buchan HA, Duggan A, Hargreaves J, Scott IA, Slawomirski L (2016). Health care variation: time to act. Med J Aust.

[8] Austin JM, Jha AK, Romano PS, Singer SJ, Vogus TJ, Wachter RM (2015). National hospital ratings systems share few common scores and may generate confusion instead of clarity. Health Aff (Millwood)..

[9] Shahian DM, Normand SL, Friedberg MW, Hutter MM, Pronovost PJ (2016). Rating the raters: the inconsistent quality of health care performance measurement. Ann Surg.

[10] Ferrell B, Connor SR, Cordes A, Dahlin CM, Fine PG, Hutton N (2007). The national agenda for quality palliative care: the National Consensus Project and the National Quality Forum. J Pain Symptom Manag.

[11] Nguyen MC, Moffatt-Bruce SD, Van Buren A, Gonsenhauser I, Eiferman DS (2018). Daily review of AHRQ patient safety indicators has important impact on value-based purchasing, reimbursement, and performance scores. Surgery..

[12] Reid PP, Compton WD, Grossman JH, Fanjiang G (2005). Crossing the Quality Chasm.

[13] Grant MJ, Booth A (2009). A typology of reviews: an analysis of 14 review types and associated methodologies. Health Inf Libr J.

[14] Tricco AC, Lillie E, Zarin W, O'Brien KK, Colquhoun H, Levac D (2018). PRISMA extension for scoping reviews (PRISMA-ScR): checklist and explanation. Ann Intern Med.

[15] McHugh ML (2012). Interrater reliability: the kappa statistic. Biochem Med (Zagreb).

[16] Hamadi H, Apatu E, Loh CA, Farah H, Walker K, Spaulding A (2019). Does level of minority presence and hospital reimbursement policy influence hospital referral region health rankings in the United States. Int J Health Plann Manag.

[17] Al-Amin M, Schiaffino MK, Park S, Harman J (2018). Sustained hospital performance on hospital consumer assessment of healthcare providers and systems Survey measures: what are the determinants?. J Healthc Manag.

[18] Yokoe DS, Avery TR, Platt R, Kleinman K, Huang SS (2018). Ranking hospitals based on Colon surgery and abdominal hysterectomy surgical site infection outcomes: impact of limiting surveillance to the operative hospital. Clin Infect Dis.

[19] Odisho AY, Etzioni R, Gore JL (2018). Beyond classic risk adjustment: socioeconomic status and hospital performance in urologic oncology surgery. Cancer..

[20] Fontana MA, Lyman S, Islam W, MacLean CH. When Stars Do Not Align: Overall Hospital Quality Star Ratings and the Volume-Outcome Association. JB JS Open Access. 2019;4(1):e0044. 10.2106/JBJS.OA.18.00044. 10.2106/JBJS.OA.18.00044 PMC6510470 31161152

[21] Lichtman JH, Leifheit EC, Wang Y, Goldstein LB (2019). Hospital quality metrics: "America's best hospitals" and outcomes after ischemic stroke. J Stroke Cerebrovasc Dis.

[22] Emmert M, Meszmer N, Schlesinger M (2018). A cross-sectional study assessing the association between online ratings and clinical quality of care measures for US hospitals: results from an observational study. BMC Health Serv Res.

[23] Frisch NB, Courtney PM, Darrith B, Della Valle CJ (2017). Do higher-volume hospitals provide better value in revision hip and knee arthroplasty?. Bone Joint J.

[24] Shapiro JM (2017). One-star rating for a five-star program: evaluating 'Hospital Compare'. Public Health.

[25] Wang DE, Wadhera RK, Bhatt DL (2018). Association of Rankings with Cardiovascular Outcomes at top-ranked hospitals vs nonranked hospitals in the United States. JAMA Cardiol.

[26] Consumer Reports [Available from: https://www.consumerreports.org/. Accessed 1 Sep 2019.

[27] The Centers for Medicare & Medicaid Services (CMS) Hospital Compare [Available from: https://www.medicare.gov/hospitalcompare/About/What-Is-HOS.htmlv. Accessed 1 Sep 2019.

[28] Healthgrades [Available from: www.healthgrades.com. Accessed 1 Sep 2019.

[29] IBM Watson Health [Available from: https://www.ibm.com/watson-health/services/100-top-hospitals. Accessed 1 Sep 2019.

[30] Why not the best IPRO [Available from: whynotthebest.org. Accessed 1 Sep 2019.

[31] The Joint Commission [Available from: https://www.jointcommission.org/. Accessed 1 Sep 2019.

[32] Leapfrog [Available from: https://www.hospitalsafetygrade.org/. Accessed 1 Sep 2019.

[33] US News and World Report [Available from: www.usnews.com. Accessed 1 Sep 2019.

[34] Walkey AJ, Shieh MS, Liu VX, Lindenauer PK (2018). Mortality measures to profile hospital performance for patients with septic shock. Crit Care Med.

[35] Hoyer EH, Padula WV, Brotman DJ, Reid N, Leung C, Lepley D (2018). Patterns of hospital performance on the hospital-wide 30-day readmission metric: is the playing field level?. J Gen Intern Med.

[36] Squitieri L, Bozic KJ, Pusic AL (2017). The role of patient-reported outcome measures in value-based payment reform. Value Health.

[37] Porter M. Value-Based Health Care Delivery. Annals of Surgery. 2008;248(4):503–09. 10.1097/SLA.0b013e31818a43af. 10.1097/SLA.0b013e31818a43af 18936561

[38] Carter J, Ward C, Wexler D, Donelan K (2018). The association between patient experience factors and likelihood of 30-day readmission: a prospective cohort study. BMJ Qual Saf.

[39] Ly A, Latimer E (2015). Housing first impact on costs and associated cost offsets: a review of the literature. Can J Psychiatr.

[40] Martin SL, Connelly N, Parsons C, Blackstone K (2018). Simply delivered meals: a tale of collaboration. Am J Manag Care.

[41] Ramon I, Chattopadhyay SK, Barnett WS, Hahn RA (2018). Early childhood education to promote health equity: a community guide economic review. J Public Health Manag Pract.

[42] Dyakova M, Hamelmann C, Bellis MA, Besnier E, Grey CNB, Ashton K (2017). Investment for health and well-being: a review of the social return on investment from public health policies to support implementing the sustainable development goals by building on health 2020.

[43] DeSalvo KBWY, Harris A, Auerbach J, Koo D, O’Carroll P. A call to action for public health to meet the challenges of the 21st century. Prev Chronic Dis. 2017;14:170017. 10.5888/pcd14.170017. PMC5590510 28880837

[44] National Health Interview Survey: National Center for Health Statistics; [Available from: https://www.cdc.gov/nchs/nhis/index.htm. Accessed 1 Sep 2019.

[45] Behavioral Risk Factor Surveillance System: Centers for DIsease Control and Prevention; [Available from: https://www.cdc.gov/brfss/index.html. Accessed 1 Sep 2019.

[46] National Health and Nutrition Examiniation Survey: National Center for Health Statistics [Available from: https://www.cdc.gov/nchs/nhanes/index.htm. Accessed 1 Sep 2019.

[47] American Community Survey: United States Census Bureau; [Available from: https://www.census.gov/programs-surveys/acs. Accessed 1 Sep 2019.

[48] Corl K, Levy M, Phillips G, Terry K, Friedrich M, Trivedi AN (2019). Racial and ethnic disparities in care following the New York state Sepsis initiative. Health Aff (Millwood)..

[49] Figueroa JF, Zheng J, Orav EJ, Epstein AM, Jha AK (2018). Medicare program associated with narrowing hospital readmission disparities between black and white patients. Health Aff (Millwood)..

[50] Bernheim SM, Parzynski CS, Horwitz L, Lin Z, Araas MJ, Ross JS (2016). Accounting for Patients' socioeconomic status does not change hospital readmission rates. Health Aff (Millwood)..

[51] Flanagan C. U.S. hospitals shut at 30-a-year pace, with no end in sight. https://www.bloomberg.com/news/articles/2018-08-21/hospitals-are-getting-eaten-away-by-market-trends-analysts-say. Accessed 1 Sep 2019.

[52] Downing NS, Cloninger A, Venkatesh AK, Hsieh A, Drye EE, Coifman RR, et al. Describing the performance of U.S. hospitals by applying big data analytics. PLoS One. 2017;12(6):e0179603. 10.1371/journal.pone.0179603. 10.1371/journal.pone.0179603 PMC5491053 28662045

[53] Bardach NS, Asteria-Peñaloza R, Boscardin WJ, Dudley RA (2013). The relationship between commercial website ratings and traditional hospital performance measures in the USA. BMJ Qual Saf.

[54] Hota B, Webb T, Chatrathi A, McAninch E, Lateef O. Disagreement between hospital rating systems: measuring the correlation of multiple benchmarks and developing a quality composite rank. Am J Med Qual. 2020;35(3):222–30. 10.1177/1062860619860250. 10.1177/1062860619860250 31253048

