# Supplementary material for: U.S. hospital performance methodologies: a scoping review to identify opportunities for crossing the quality chasm
Source: BMC Health Serv Res. 2020 Jul 10;20:640. doi: 10.1186/s12913-020-05503-z (PMC7350649; doi:10.1186/s12913-020-05503-z)
Supplement: Supplementary file 1 — Additional file 1. [file 12913_2020_5503_MOESM1_ESM.docx]

**Additional File 1**

**Methods description**

The scoping review protocol was executed to identify methodologies used for the purpose of reviewing hospital performance in the United States. A search query (Table 1) was used to identify published literature from Medline via PubMed. Handsearching of web-published content was also performed using facets from the search query as many commercially available hospital performance assessments are not peer-reviewed.

One reviewer screened all titles and abstracts from Medline and web-content was examined by two independent reviewers for relevance against the inclusion/exclusion criteria (Table 2). Content marked for relevance were examined by two independent reviewers at the level of full-text and third-party adjudication was provided for any discrepancies in eligibility. Results were tracked in DistillerSR (Evidence Partners).

Included publications or web-based content was dually reviewed. Data was abstracted into standardized forms (See Additional File 2) for synthesis and evaluation by a single reviewer and the content was examined for quality and completeness by a second reviewer. Conflict resolution was provided by a third-party reviewer, if necessary.

**Tables**

*Table 1. Medline search via PubMed*

| **Search no.** | **Facet** | **Search Terms** | **Search Results**  **(September 19, 2019)** |
| --- | --- | --- | --- |
| 1 | Hospital assessments | hospital AND (rating OR ranking OR list) | 70,302 |
| 2 | Hospital performance | quality OR performance OR access OR utilization OR efficiency | 5,024,152 |
| 3 | Methodology | method* | 7,252,929 |
| 4 | Combine | #1 AND #2 AND #3 | 19,106 |
| 5 | Limit to Publication Date | #4 Filter: published September 1, 2017 – September 1, 2019 | 4,237 |
| 6 | Limit to Studies with Abstracts | #4 Filters: Published in date of interest; Abstract | 4,229 |
| 7 | Limit to English | #4 Filters: Published in date of interest; Abstract; English | 4,075 |
| 8 | Limit to studies in humans | ##4 Filters: Published in date of interest; Abstract; English; Humans | 3,043 |

*Table 2. Inclusion/Exclusion Criteria*

Articles will be considered eligible for the scoping review if they meet the following inclusion/exclusion criteria:

| **Inclusion Criteria** | **Exclusion Criteria** |
| --- | --- |
| The publication regards humans. | The publication does not regard humans. |
| The publication is in English. | The publication is in a language other than English. |
| The publication contains an abstract. | The publication does not contain an abstract. |
| The publication date is within September 1, 2017 to September 1, 2019. | The publication date is before September 1, 2017 or after September 1, 2019. |
| The publication provides an explicit methodology to examine hospital performance (quality, access, efficiency, or utilization). | The publication does not provide an explicit methodology to examine hospital performance (quality, access, efficiency, or utilization) or study does not provide enough information to replicate design and execution of their evaluation methods.  The performance assessment is based on nominations or applications rather than using publicly available data for evaluation. |
| The publication provides a hospital assessment that compares > 250 hospitals (any type, any ownership). | The publication provides an assessment of <250 hospitals. |
| The publication describes hospital performance in the United States. | The publication describes hospital performance outside of the United States. |

Footnote: Publications or web references were considered.

Table 3. Abstraction Tables for Included Studies

Abstraction tables can be found in abstractions.zip of Additional File 2.

**Results**

*Assessment data sources*

Data sources used for assessment generally fell into survey, claims, or registry data, and most assessments used some combination of these sources. Across assessments, outcomes data was primarily leveraged, mostly mortality and patient safety-related data (e.g., hospital-acquired infections). Patient experience measures such as the Consumer Assessment of Healthcare Providers and Systems survey (HCAHPS), cost data from sources like Healthcare Cost Report Information System (HCRIS), and structural resource data such as number of doctors and their level of expertise (e.g. board certifications) were all additional types of measures used for assessment.

Data was obtained across both the public and private sectors. Mostly, publicly available data sources were leveraged, such as Medicare Provider Analysis and Review (MedPAR) and HCRIS datasets. The most commonly used public data source across assessments was the CMS Hospital Compare dataset. Second to public sources, private data was used, for example survey data from the American Hospital Association (AHA) regarding health information technology usage or registry data, such as Doximity’s information on provider expertise and training certifications. The Joint Commission was the only performance assessment using solely private sources of data.

On average, a total of between five and six data sources were used, with the minimum being one (Healthgrades) and the maximum being 17 (IPRO). No assessments provided information on how data was specifically linked to other datasets or aggregated, at least in enough detail to be replicated. The temporal range of data used for assessment varied based on these data sources, but generally data was collected for one year previous to the time of an assessment being conducted. Assessments that included CMS Hospital Compare data spanned the longest timeframes, across approximately three years. Within each data source the amount of data was highly variable ranging in size from 250 hospitals derived from MedPAR data to 4,746 hospitals for the CMS Hospital Compare dataset.

*Cohort development*

Most assessments had criteria for selecting hospitals to include based on the availability of data (*Consumer Reports*, Healthgrades, Hospital Compare, IBM, Leapfrog, *US News*). Age and clinical characteristics of populations served were also used to select hospitals for assessment (*Consumer Reports*, IBM, *US News*). Otherwise, little information existed on how hospitals were selected for assessment (Joint Commission). Data pre-processing information was also limited. Mostly, assessments described how outliers were identified (IBM, IPRO, Leapfrog), explained how boundary checks or verification on temporal and conceptual elements of their data were performed (*Consumer Reports*), or how data was aggregated across datasets (*Consumer Reports).*

*Score*

Most scoring across assessments combined many measures into one "overall" score, even though the individual measures may have addressed quite different aspects of quality; examples included *Consumer Reports*, Hospital Compare, IBM, IPRO, Leapfrog, *US News*.

To determine the weights for these different indicators, latent variable modeling approaches (Hospital Compare) were used to estimate quality including methods like confirmatory factor analysis (*US News*). Some assessments assigned pre-determined weights based on expert-driven criteria (Healthgrades, Leapfrog), weighted or standardized averages (*Consumer Reports*, IBM), or percent of time recommended care was provided (Joint Commission). One assessment, IPRO, did not specify any weighting for the measures assessed.

Once the weights for different indicators or measures were identified, ratings were assigned to groups via: (1) clustering (Hospital Compare), (2) weights based on expert consensus (*US News*), (3) selecting the top percentile (Healthgrades, IPRO), or (4) summing across measure types (*Consumer Reports*, IBM, Leapfrog) and then normalizing to a scale. Some assessments did not calculate an overall score (Joint Commission).
